# The Influence of Stress on the Transition From Drug Use to Addiction

**Published:** 2008

**Authors:** Gary Wand

**Keywords:** Addiction, alcohol and other drug (AOD) dependence, stress, stress as an AOD cause (AODC), stress response, brain, brain reward pathway, glucocorticoid, dopamine, animal studies, human studies

## Abstract

Stress—that is, any type of stimulus that challenges the organism’s normal internal balance—induces a physiologic response involving a variety of hormones and other signaling molecules that act on, among other organs, the brain. This stress response also can influence the progression of alcohol and other drug (AOD) addiction through various stages. For example, AODs can directly activate the stress response. In turn, certain stress hormones (i.e., glucocorticoids and corticotrophin-releasing factor) also act on the brain system that mediates the rewarding experiences associated with AOD use (i.e., the mesocorticolimbic dopamine system). Moreover, elevated glucocorticoid levels and stress increase AOD self-administration in certain animal models. During a later stage of the addiction process, in contrast, excessive and/or prolonged stress may impair the reward system, inducing heavier AOD use to maintain the rewarding experience. During the final stage of addiction, when the addicted person experiences withdrawal symptoms if no drug is consumed, chronic AOD use results in gross impairment of the normal stress response and other signaling mechanisms in the brain, resulting in a state of anxiety and internal stress. At this stage, people continue to use AODs mainly to relieve this negative-affect state.

Addiction to alcohol and other drugs (AODs) is a complex phenomenon influenced by genetic and environmental determinants. For example, in both animals (i.e., rodents and nonhuman primates) and humans, various forms of stress play a role in escalating AOD use as the individual progresses from episodic exposure to addiction. Studies in specific rodent models have shown that stress and certain hormones released in response to stress (i.e., glucocorticoids^1^) increase AOD self-administration during the earliest stage of addiction development (i.e., the acquisition phase). (The stages of AOD addiction development are discussed in more detail in the section “Theories of Addiction.”) Once rodents are addicted to AODs, high levels of glucocorticoids and other stress-related molecules (i.e., stress peptides) produced by the addicted animal create an internal form of stress that is characterized by anxiety-like behaviors and dysfunction of the brain’s reward system. This negative-affect state further escalates AOD consumption.

In humans, numerous stressors increase the risk of alcohol use disorders (Richman et al. 1996; Rospenda et al. 2000; Vasse et al. 1998). In AOD-dependent people, internal and external forms of stress increase drug craving (Sinha et al. 2003) and may trigger relapse (Sinha et al. 2006). This article explores how various forms of stress may contribute to AOD use as the individual transitions from occasional AOD use to dependence. After providing some general information on stress and on theories of addiction, the article summarizes the findings of animal and human models examining the detrimental effects of stress and stress hormones on the brain’s reward pathways that mediate the pleasurable or rewarding effects of drinking. These effects ultimately result in a dysfunction of the reward system and contribute to escalation of AOD use.

## What Is Stress?

Stress generally is defined as any stimulus that challenges physiological homeostasis—that is, which alters the balance or equilibrium of the normal physiological state of the organism. It is important to realize, however, that the term “stress” is rather nonspecific and should always be qualified. Although all forms of stress alter homeostasis, they do not all do so in the same manner (i.e., they have different physiological consequences). Different kinds of stress can stimulate different combinations of signaling molecules (i.e., molecules that aid in cell-to-cell communication, such as neurohormones), thereby producing unique effects on physiological processes. Accordingly, it is imperative to specify the type and duration of stress to which an organism is subjected. Moreover, reactions to a given type of stress vary between individuals, and physiological and behavioral responses tend to be associated with distinct coping styles (Øverli et al. 2007).

Like the susceptibility to AOD use disorders, people’s responses to stress are regulated by the interaction of environmental and genetic factors. In fact, prenatal and early-life stress can have lifelong effects on the body systems involved in the stress response, and these effects may predispose an individual to certain diseases (Maccari et al. 2003). This early programming effect in part is influenced by mechanisms (i.e., epigenetic mechanisms) that alter heritable traits without manifesting as changes in the DNA sequence and which also can aid in the development of AOD use disorders.

### The Stress Response

Mammals respond to the various forms of stress with a response comprising at least three components:

- The glucocorticoid response mediated through a hormone system known as the hypothalamic–pituitary–adrenal (HPA) axis;
- Activation of the peptide corticotrophin-releasing factor (CRF) outside the hypothalamus; and
- Activation of one branch of the nervous system (i.e., the sympathetic nervous system) that results in the release of signaling molecules called epinephrine (or adrenaline) and norepinephrine (or noradrenaline).

**Figure 1:**
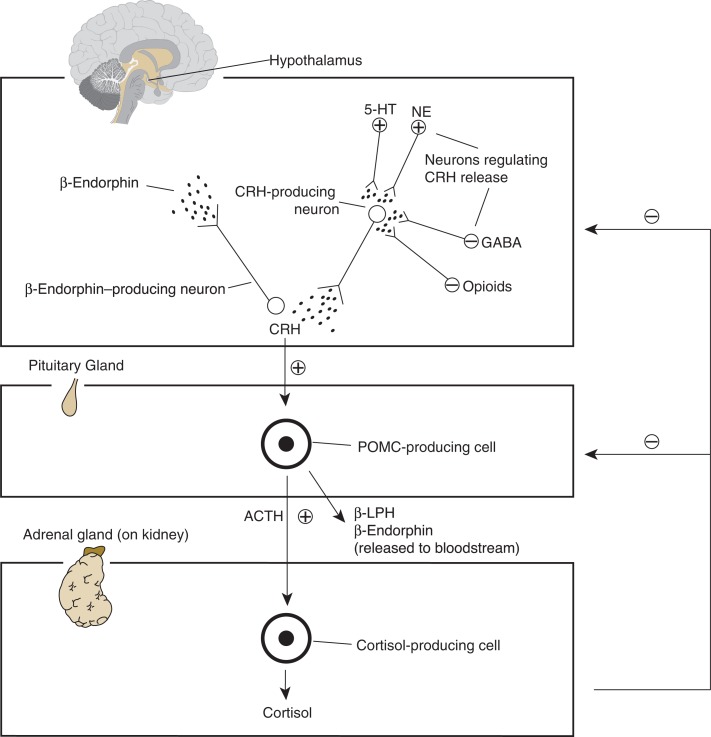
The major components of the stress response mediated by the hypothalimic-pituitary-adrenal (HPA) axis. Both alcohol and stress can induce nerve cells in one brain region (i.e., the hypothalamus) to produce and release corticotropin-releasing hormone (CRH). Within the hypothalamus, CRH stimulates the release of a hormone that produces morphine-like effects (i.e., β-endorphin). CRH also is transported to a key endocrine gland, the anterior pituitary gland. There, CRH stimulates production of a protein called proopiomelanocortin (POMC). POMC serves as the basis for a number of stress-related hormones, including adrenocorticotropic hormone (ACTH), β-lipotropin (β-LPH), and β-endorphin. ACTH stimulates cells of the adrenal glands to produce and release the stress hormone cortisol. When cortisol levels reach a certain level, CRH and ACTH release diminishes. Other neurons releasing serotonin (5-HT), norepin-ephrine (NE), γ-aminobutyric acid (GABA), or endogenous opioids also regulate CRH release. NOTE: ⊕ = excites; ⊖ = inhibits.

The glucocorticoid response mediated by the HPA axis involves, as the name implies, three distinct organs (see figure 1):

- The hypothalamus, a brain region located deep within the brain;
- The pituitary gland, a small, hormone-secreting gland located directly below the hypothalamus; and
- The adrenal glands, hormone-producing structures located on top of the kidneys.

When an individual is in a stressful situation, the hypothalamus releases CRF. This hormone then is transported to a certain region of the pituitary gland (i.e., the anterior pituitary), where it interacts with specific proteins on the surface of the pituitary cells (i.e., CRH-1 receptors).^2^ This interaction stimulates the anterior pituitary to produce a molecule called proopiomelanocortin (POMC), which then is processed further into smaller, biologically active peptides, including β-endorphin and adrenocorticotropic hormone (ACTH). The ACTH subsequently is carried via the blood to the adrenal glands, where it induces secretion of glucocorticoids. The main glucocorticoid in humans and other primates is cortisol; the main glucocorticoid in rodents is called corticosterone. The glucorticoids are transported by the blood to the brain, where they act on numerous signaling systems and also on the AOD reward system, as well as to numerous other organs throughout the body, where they induce some of the physiological stress responses (e.g., increases in blood glucose levels and blood pressure).

The activity of the HPA axis is regulated by a sensitive negative-feedback loop. This means that the glucocorticoids secreted by the adrenal glands are transported through the bloodstream back to the pituitary gland, hypothalamus, and hippocampus, where they interact with specific receptors to shut off the HPA axis and thereby limit the stress response. There are different types of glucocorticoid receptors:

- Type I mineralocorticoid receptors tightly bind to cortisol even at low cortisol concentrations (i.e., have a high affinity for cortisol); as a result, these receptors usually interact with cortisol at the low levels normally found in the body, thereby helping to regulate HPA axis activity in the normal, resting state.
- Type II glucocorticoid receptors have a lower affinity for cortisol and only interact with it when cortisol levels in the blood are elevated during stressful situations. Accordingly, these receptors primarily help regulate the stress response.

The interaction of glucocorticoids with both types of receptors leads to the activation of certain signaling molecules in the cells (i.e., transcription factors) that, in turn, activate certain genes; however, the two types of receptors activate different sets of genes.

CRF is produced not only in the hypothalamus but also in other brain regions, and stress also stimulates the production of this CRF. This extrahypothalamic CRF has three effects: First, it stimulates the HPA axis, just like the CRF produced by the hypothalamus. Second, it activates the sympathetic nervous system, the branch of the nervous system that is primarily responsible for regulating many homeostatic mechanisms in living organisms. For example, the sympathetic nervous system coordinates the neuronal and hormonal response to stress (the “fight-or-flight” response). In a stressful situation, the sympathetic nervous system causes the release of epinephrine and norepinephrine from the adrenal glands. These hormones help prepare the body for an emergency situation. For example, both epinephrine and norepinephrine increase the supply of oxygen and nutrients to the brain and muscles (e.g., by increasing heart rate and widening blood vessels in the muscle). Moreover, these hormones suppress nonessential bodily processes (e.g., digestion). Finally, norepinephrine also acts on the brain to increase attention and promote responding actions.

**Figure 2:**
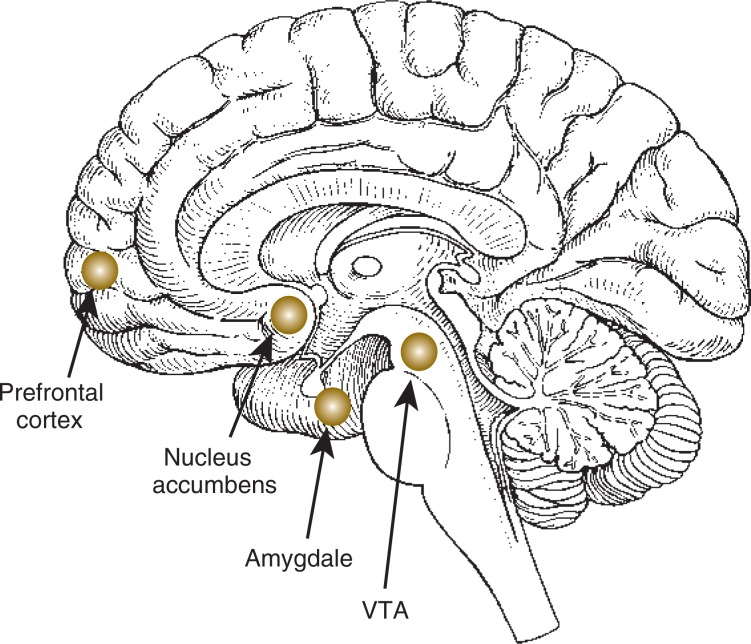
Location of the components of the mesocorticolimbic dopamine system and other brain regions affected by the stress response and its interactions with alcohol and other drugs.

Third, increased CRF production outside the hypothalamus stimulates a brain system known as the mesocorticolimbic dopamine system. This brain pathway includes several interconnected brain areas whose activity involves the signaling molecule (i.e., neurotransmitter) dopamine. The components of this system are the ventral tegmental area (VTA), the nucleus accumbens, and the constituents of the limbic system (particularly the hippocampus and amygdala), all of which are located deep inside the brain, as well as some areas of the outer layer of the brain (i.e., the prefrontal cortex) (see figure 2). (The mesocorticolimbic system will be described in more detail later in this article.) The mesocorticolimbic dopamine system represents the brain’s reward pathway—that is, it mediates the rewarding effects associated with AOD use and other experiences and thereby is a central factor in the development of addiction. In addition, the hypothalamus and limbic system, particularly the hippocampus and amygdala, are intimately involved in the stress response (e.g., anxiety and other stress behaviors) that is initiated by the stimulation of CRF activity.

### Allostasis

Allostasis is the process utilized by mammals to maintain homeostasis through a variety of physiological or behavioral changes when the organism is threatened by various forms of stress. It involves a series of dynamic actions through which the hormones of the HPA axis, immune factors (i.e., cytokines), and signaling molecules used by the autonomic nervous system are triggered (McEwen 2007). When an organism is exposed to extended periods of stress, it is said to be accumulating an allostatic load. If the stressful situation persists, the allostatic load increases, resulting in wear and tear on the organism from excessive exposure to glucocorticoids, stress peptides, and inflammation-promoting (i.e., proinflammatory) cytokines. For example, chronic activation of the HPA axis is associated with the development of mood and anxiety disorders, such as depression (Gillespi and Nemeroff 2005). Likewise, excess cortisol exposure can contribute to various medical problems, including obesity, insulin resistance, bone demineralization, cognitive impairment, and impaired immunity (Kyrou et al. 2006; Lee et al. 2007).

As described in more detail in the following section, the concept of allostasis also is used in addiction research. In this context, the term “allostatic load” refers to a state of reward dysregulation—that is, a state in which the individual’s drug-seeking behavior is driven by a need to recapture the initial rewarding effects of the drug (i.e., “chasing the high”). With increasing allostatic load, environmental events or cues that previously have been associated with drug use become increasingly important (i.e., have increased salience) to the addicted individual.

## Theories of Addiction

Many theories of addiction have been proposed to explain mechanisms involved in this complicated disorder. This review focuses on two of these models—incentive sensitization and hedonic allostasis—that can be combined to conceptualize the impact that various forms of stress have on the development of drug dependence (Vanderschuren and Everitt 2005). According to the incentive sensitization model, in some people the effects of casual AOD use sensitize the mesocorticolimbic reward system so that the drug user wants to repeat these rewarding effects. This results in an extremely high motivation to use AODs again. This model helps explain how initial experimentation with AODs by an occasional user can escalate to repeated drug administration. The motivational force at this stage is primarily hedonic pleasure. With chronic AOD use, how ever, the motivation for drug seeking can change as a result of hedonic allostasis. According to the hedonic allostasis model, chronic AOD exposure results in reduced activity (i.e., downregulation) of positive reward circuits, which generates a chronic stress situation. As a result, the body produces stress factors that create a negative emotional state (i.e., negative affect) characterized by withdrawal symptoms, sadness (i.e., dysphoria), and anxiety. This negative affect then becomes the dominant force driving drug craving. Thus, the individual no longer seeks to use AODs to achieve a pleasurable experience but to avoid a negative-affect state. Various forms of stress can impact this transition from incentive sensitization to hedonic allostasis.

Koob and Le Moal (1997) have proposed that drug addiction progresses through three stages:

- The preoccupation/anticipation stage, which is characterized by exaggerated motivation for drug use associated with a sensitized mesocorticolimbic dopamine system. This phase is modeled by the incentive sensitization theory. In animal models used to study the development of AOD dependence, this stage is referred to as the acquisition phase.
- The binge/intoxication stage, during which a downregulation of positive reward pathways occurs—that is, the drug levels needed to trigger the brain reward system increase (Koob and Kreek 2007). This corresponds to the maintenance phase in animal models.
- The withdrawal/negative-affect stage, during which the drug user transitions to drug addiction. During this stage, negative affect becomes dominant, further escalating craving and use of the addictive drug. The analogous state in animal models can be created by experimental designs in which the drug is withheld from the animals (i.e., using drug deprivation protocols).

There is a harmful interaction between AODs and the stress response that influences the transition through these three stages of addiction. AODs can “hijack” the stress and reward systems in three ways. First, AODs directly activate the stress response, and the glucocorticoids released during this process can, at least initially, sensitize the reward pathways, leading to further AOD use. This may contribute to reward sensitization during the preoccupation stage. Second, as the AOD user transitions to addiction, negative affect and withdrawal symptoms become a dominant force that also stimulates the release of anxiety-inducing (i.e., anxiogenic) stress peptides such as CRF. At this stage, AOD use escalates as the addict administers the AODs more for stress-dampening purposes than for pleasure. Third, environmental stress (which often is caused or exacerbated by the chaotic lifestyle and negative consequences of AOD abuse) interacts with the negative-affect state created by withdrawal or abstinence to amplify the anxiety and dysphoria induced by the stress peptides. At each of these steps, allostatic mechanisms are initiated by the organism to try and maintain homeostasis in the presence of the AODs and the resulting stress.

The following sections review the evidence supporting the relationship between various forms of stress and AOD use disorders as it fits into this model of allostatic transition from reward sensitization to negative-affect development (Koob and Kreek 2007). Where appropriate, these processes will be discussed in the context of the proposed three stages of addiction (Koob and Le Moal 1997). The discussion primarily focuses on alcohol and cocaine, whose relationship with stress has been studied best.

## Stage One: AOD Use Based on Positive Reward

### The Mesocorticolimbic Dopamine System and Reward Pathways

As mentioned earlier, the mesocorticolimbic dopamine system functions as a reward and reinforcement pathway, providing salience to internal and external stimuli (Berridge 2007). In other words, this system governs our attention and intentions related to a particular stimulus, and its integrity is crucial for maintaining a healthy response to stimuli.

The central components of the mesocorticolimbic dopamine system are nerve cells (i.e., neurons) whose cell bodies are located in a region called the ventral tegmental area (VTA) (see figure 2). From these cell bodies, long extensions (i.e., axons) reach out to various other brain regions, most prominently the nucleus accumbens (NAc), which is located in a brain region called the ventral striatum and is part of the limbic system, and the prefrontal cortex. The NAc is thought to assign importance (i.e., salience) to the drug experience. In the NAc and prefrontal cortex, the VTA neurons release the neurotransmitter dopamine from the ends of their axons into the space separating the axon from the neighboring cell (i.e., into the synapse). The released dopamine then interacts with specific receptors on the neighboring neurons in those brain regions, thereby altering their activity in a specific manner.

Cells of the mesocorticolimbic dopamine system have several properties that allow them to mediate the rewarding experience associated with AOD use and other events. For example, the dopaminergic neurons can transmit signals either by emitting short bursts of several signals, which is known as phasic firing, or by emitting low signals over longer periods of time, which is known as tonic firing (Grace 2000). The particular patterns of tonic and phasic firing may determine the salience of the reward signal (Grace 2000). Another characteristic property of the mesocorticolimbic dopamine system is its ability to generate signals that represent the relationship between the rewarding experience a drinker has come to expect after consuming a certain amount of alcohol (i.e., the predicted reward) and the actual reward associated with a particular experience (Schultz et al. 1997)—a reward that is greater than predicted results in increased firing of the dopamine-releasing (i.e., dopaminergic) neurons and, conversely, a reward that is less than predicted results in decreased firing of these neurons. Rewards that are as predicted do not alter firing rates. Some researchers have proposed that during the transition from recreational AOD use to addiction, the reward signal associated with drug use shifts from “better than predicted” to “worse than predicted” (Schultz et al. 1997).

AODs have both positive and negative reinforcing properties. During the first stage of the progression from AOD use to addiction, the positive reinforcing properties, which are linked to the hedonic aspects of drug intoxication, prevail. Considerable evidence shows that these positive reinforcing effects are mediated by signal transduction systems that stimulate the mesocorticolimbic dopamine neurons. Thus, casual use of alcohol, psychostimulants, and opioids increases dopamine accumulation in the synapses within the NAc and other affected regions and thereby provides salience to their signal (Spanagel and Heilig 2005).

### Animal Studies on Stress, Glucocorticoids, and Mesocorticolimbic System Function

**Figure 3:**
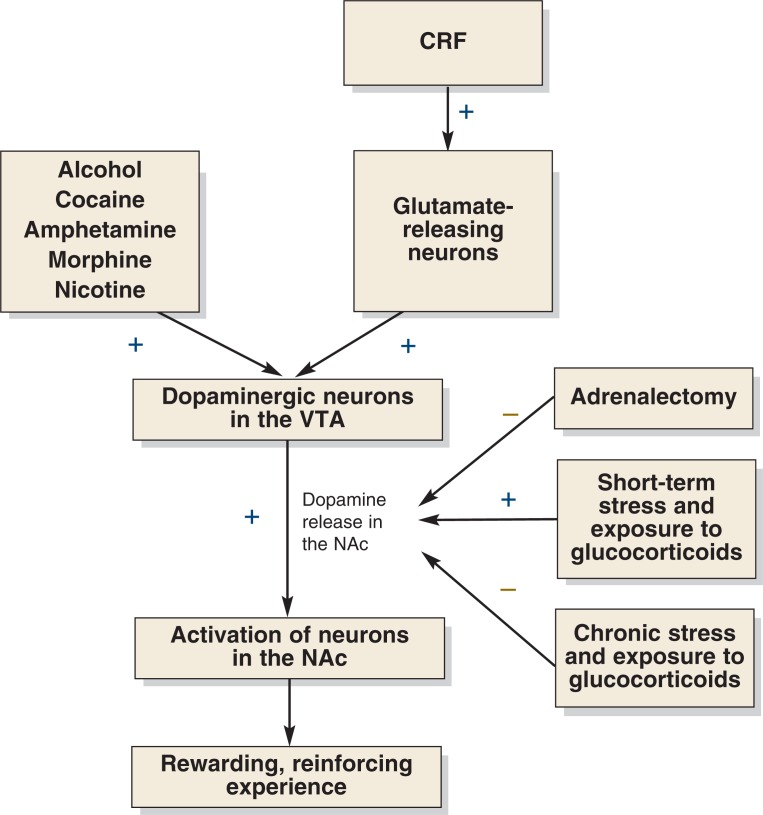
Regulation of the mesocorticolimbic dopamine system. Cell bodies of dopamine-releasing (i.e., dopaminergic) neurons located in the ventral tegmental area (VTA) are activated by glutamate-releasing neurons. This activation leads to the release of dopamine in the nucleus accumbens (NAc), resulting in the activation of other neurons whose cell bodies are located in the NAc and in the generation of rewarding and reinforcing experiences. Alcohol and other drugs (AODs), corticotrophin-releasing factor (CRF), and stress and stress-induced hormones (i.e., glucocorticoids) all can influence this chain of events by acting on the glutamate-releasing neurons, dopaminergic neurons in the VTA, or dopamine release in the NAc.

As mentioned earlier, the ultimate result of the body’s stress response via the HPA axis is the release of glucocorticoids from the adrenal glands. Therefore, to investigate if and how stress can influence the perceived effects of AODs, one approach is to study, in laboratory animals, how glucocorticoids alter mesocorticolimbic signaling and thereby modulate (e.g., amplify) the positive reinforcing effects of AODs. For example, researchers lowered animals’ glucocorticoid concentrations in the blood by removing the animals’ adrenal glands (i.e., performing an adrenalectomy). This manipulation led to reduced dopamine levels in the NAc, both when the VTA neurons were in a resting state and when they were stimulated (Barrot et al. 2000; Piazza et al. 1996) (see figure 3). When the animals were injected with corticosterone to replace adrenal glucocorticoid production, normal dopamine levels were restored. Additional analyses demonstrated that the effects of adrenalectomy on dopamine levels were limited only to certain regions of the NAc (i.e., the shell of the NAc) (Barrot et al. 2000). Furthermore, corticosterone’s effects on dopamine concentrations in the shell of the NAc involve activation of the glucocorticoid (type II) receptors, which only are activated at higher corticosterone concentrations but not the mineralocorticoid (type I) receptors, which also are activated at lower corticosterone concentrations (Marinelli and Piazza 2002).

Many studies have demonstrated that not only glucocorticoid manipulation but also stress will increase dopamine levels in the NAc. For example, using microdialysis techniques, investigators have shown that diverse experiments involving different types of stress can increase mesocorticolimbic dopamine activity (Marinelli and Piazza 2002). Other studies, however, do not support these findings (Imperato et al. 1989, 1991; Reid et al. 1998). Additionally, one study (Pacak et al. 2002) showed that chronic exposure to elevated corticosterone levels inhibits dopamine synthesis and turnover, suggesting that the effects of glucocortiocoids on the mesocorticolimbic dopamine system depend on the duration of glucocorticoid exposure. Thus, short-term exposure to glucocorticoids increases dopamine levels, whereas chronic exposure results in decreased dopamine levels.^3^ Interestingly, elevated glucocorticoid levels resulting from exposure to stressors or injections of corticosterone also can sensitize animals to the behavioral and neurochemical responses (e.g., dopamine release in the NAc) to cocaine (Prasad et al. 1998; Rouge-Pont et al. 1995). Conversely, these effects are attenuated in rats that no longer produce corticosterone (Prasad et al. 1998; Przegalinski et al. 2000; Rouge-Pont et al. 1995). Thus, it appears that glucocorticoid concentrations in the normal range are necessary for optimal transmission of dopamine-mediated signals in the mesocorticolimbic system.

Another important region of the mesocorticolimbic system is the VTA, where the bodies of the dopaminergic axons ending in the NAc originate. To trigger dopamine release by these neurons in the NAc, nerve signals emitted by other neurons must activate (i.e., excite) the bodies of the dopaminergic neurons in the VTA. Various drugs (e.g., cocaine, amphetamine, morphine, nicotine, and alcohol) as well as CRF administration increase excitatory synaptic strength in the VTA (Kauer 2003) (see figure 3). This means that after a drug or CRF is administered, neurons that act on the cell bodies of the dopaminergic neurons in the VTA send out more and/or stronger signals, thereby leading to increased excitement of the dopaminergic neurons. In addition, the dopaminergic neurons in the VTA can be activated by corticosterone (e.g., after exposure to a stressful situation). For this activation to occur, other neurons must emit a signal by releasing the neurotransmitter glutamate, which then can interact with receptors on the dopaminergic cells (Overton et al. 1996). Administration of an agent that inhibits these receptors (i.e., the *N*-methyl-d-aspartic acid [NMDA] receptor antagonist MK-801) blocks stress-induced changes in glutamate-mediated signal transmission (Morrow et al. 1993). Moreover, administration of a glucocorticoid receptor antagonist before stress also prevents the synaptic changes. Finally, when laboratory animals were exposed to a stressful situation, the resulting stress activated CRF in the VTA and thereby stimulated glutamate-mediated signal transmission to dopaminergic neurons (Wang et al. 2005). Taken together, these findings suggest that CRF and glucocorticoids released during stress are responsible for enhancing glutamate-mediated signaling, ultimately resulting in increased dopamine release in the NAc.

In summary, stress and stress-induced glucocorticoids appear to influence the mesocorticolimbic dopamine system through two separate actions: First, they lead to the activation of the cell bodies of the dopaminergic neurons in the VTA, primarily through stimulation of glutamate-mediated signaling. This indirectly leads to increased dopamine release in the NAc. Second, they directly impact the axons of the dopaminergic neurons in the NAc, thereby further enhancing dopamine release in that brain region.

### Animal Studies on the Effects of Glucocorticoids and Stress on AOD Self-Administration

#### Psychostimulants

Animal studies demonstrated that the acquisition stage—the first time that an animal comes into contact with a drug and its rewarding properties—is modulated by glucocorticoids. For example, when investigators reduced glucocorticoid levels in the blood through both surgical and pharmacologic manipulation, the animals stopped cocaine self-administration during acquisition (Goeders 2002). This reduction in self-administration could be reversed in a dose-dependent manner by administering different amounts of external corticosterone.

Acquisition also is a time when the mesocorticolimbic system can be sensitized. The contribution of corticosteroids to this sensitization has been demonstrated in several ways. For example, during the acquisition stage, cocaine self-administration activates the HPA axis, leading to increased corticosterone levels in the blood in rodents (Goeders 2002). Other studies demonstrated that repeated administration of glucocorticoids at doses typically found during stressful situations increases drug self-administration (Goeders 2002). Moreover, glucocorticoid administration can facilitate the psychomotor stimulant effects of cocaine (Marinelli et al. 1994), amphetamine (Cador et al. 1993), and morphine (Marinelli et al. 1994).

Another strategy to investigate how increased glucocorticoid levels affect AOD self-administration is to expose the animals to stressful situations that activate the HPA axis. Multiple studies have shown that various experimental procedures to induce different types of both short-term (i.e., acute) and long-term (i.e., chronic) stress increase psychostimulant or opiate self-administration in rats (Marinelli and Piazza 2002). Exposure to electric footshock also increases the subsequent reinforcing effects of heroin in rats (Shaham and Stewart 1994).

In another series of studies, researchers further explored the role of glucocorticoids in cocaine self-administration during the acquisition, maintenance, and reinstatement periods in order to better understand the conditions required for glucocorticoids and stress to increase cocaine self-administration (Goeders 2003). During the acquisition phase, rats exposed to stressors (e.g., electric shocks) they could not control exhibited increased rates of low-dose (but not high-dose) cocaine self-administration compared with rats exposed to no stressors or to stressors they could control through their behavior. Moreover, self-administration did not occur unless corticosterone levels in the blood were increased above a specific threshold, and greater stress-induced increases in corticosterone levels were associated with greater increases in self-administration (Goeders 2002). The same effects could be achieved if corticosterone levels were increased directly by administering the hormone rather than indirectly by exposing the animals to a stressor. Importantly, this effect occurred only during the acquisition phase, not during the maintenance phase or during cue-induced reinstatement of cocaine self-administration. These findings were confirmed by other investigators who demonstrated that blood corticosterone levels predicted how much cocaine the animals would self-administer (Koob and Kreek 2007; Mantsch et al. 2001) and that this relationship was only observed at low doses of cocaine (Mantsch et al. 2000). Together, these findings indicate that individual differences in stress-related cocaine self-administration are relevant only with low doses of cocaine and only during the acquisition period.

As mentioned earlier, corticosterone binds to both mineralocorticoid receptors (which bind to corticosterone at normal, low concentrations) and glucocorticoid receptors (which bind to corticosterone only at high, stress-induced concentrations). The relationship between stress and cocaine self-administration appears to involve only the glucocorticoid receptors. For example, administration of an agent that specifically inactivates the glucocorticoid receptor (i.e., the glucocorticoid receptor antagonist RU486) reduces cocaine self-administration in rats (Deroche-Gamonet et al. 2003). Moreover, when the investigators used genetic engineering techniques to create mice that produced no glucocorticoid receptors, these animals self-administered less cocaine than did normal mice (Deroche-Gamonet et al. 2003). Sensitization to cocaine’s psychomotor effects also was suppressed in the modified animals. This observation was confirmed by other researchers using a different set of genetically modified animals in whom the activity of the genes encoding the glucocorticoid receptor (i.e., the levels of glucocorticoid receptor mRNA) was reduced by 50 percent (St. Hilaire et al. 2003). Other studies, in contrast, found that reduction of glucocorticoid receptor mRNA actually stimulated the mesocorticolimbic dopamine system (Cyr et al. 2001; Steckler and Holsboer 2001). Nevertheless, the prevailing evidence suggests that stress modulates low-dose cocaine self-administration through actions at the glucocorticoid receptor.

#### Alcohol

In rodents, acute alcohol administration, like psychostimulant administration, activates the HPA axis by inducing CRF release from the hypothalamus (Lee et al. 2004). However, the effects of glucocorticoids and stress during the acquisition phase of alcohol exposure have not been studied as systematically as during the acquisition phase for psychostimulants. Moreover, studies designed to examine the effects of various forms of stress on alcohol consumption in nondependent rodents have produced less consistent results than models using alcohol deprivation of dependent animals (see below), with nondependent animals showing highly variable stress-induced increases in alcohol consumption.

Finally, it appears that in rodents, the effect of stress on voluntary alcohol consumption is influenced both by the type of stress and by the genetic background of the animal. For example, stress caused by electric shocks increased alcohol consumption in several different lines and strains of non–alcohol-dependent rodents (i.e., unselected Wistar rats, alcohol-preferring [P] rats, high-alcohol–drinking [HAD] rats, and Alko alcohol [AA] rats) (Vengeliene et al. 2003). Another type of stress (i.e., swim stress), however, increased alcohol consumption only in the unselected Wistar rats, not in other strains or lines. Similar differences in the response to stressful situations were observed in different strains of mice. Finally, chronic, unpredictable restraint stress actually reduced voluntary alcohol drinking during the application of stress in P rats and HAD1 rats (Chester et al. 2004).^4^

### The Relationship of Stress, Glucocorticoids, and AOD Use in Humans

#### Effects of Acute Alcohol and Cocaine on the HPA Axis and Mesocorticolimbic Dopamine

Like various forms of stress, alcohol and psychostimulant consumption can lead to increased glucocorticoid levels in humans. Alcohol drinking in humans stimulates the HPA axis as it does in rodents. However, the response is not clearly dose dependent and most likely is observed when blood alcohol concentrations are greater than 0.1 percent (Wand 1993). Psychostimulants such as intravenous, intranasal, and smoked cocaine also activate the HPA axis, increasing the secretion of cortisol (Mello and Mendelson 1997).

If the findings obtained in rodents also reflect the conditions in humans, then increased glucocorticoid levels associated with initial stress and/or alcohol or psychostimulant use may also sensitize mesocorticolimbic reward pathways in humans. This also would mean that changes in glucocorticoid levels can alter the rewarding experiences associated with AOD use during the early stage of use. The next section reviews the evidence that a relationship between cortisol, mesocorticolimbic dopamine, and positive subjective feelings about or responses to the drug (i.e., drug liking) exists in social (i.e., nonaddicted) drinkers.

### Relationship Between Cortisol, Mesocorticolimbic Dopamine, and Drug Liking

Imaging methods that can show receptor activity in the brain, such as positron emission tomography (PET) imaging, allow researchers to translate observations originally made in rodent models to humans and to study the effect of AODs on mesocorticolimbic dopaminergic activity. For example, PET imaging studies on the effects of alcohol, amphetamine, methylphenidate, and cocaine found that mesocorticolimbic dopamine responses are correlated with positive subjective effects of the drug that originally triggered dopamine release (Boileau et al. 2003; Oswald et al. 2005; Volkow et al. 1999). These and other studies corroborate the results of animal studies demonstrating that AODs activate mesocorticolimbic dopamine.

Researchers also have used PET technology to study the relationship among stress hormones, mesocorticolimbic dopamine activity, and drug liking. For example, Pruessner and colleagues (2004) showed that in people who reported receiving insufficient maternal care during their early lives, dopamine release in the ventral striatum, which also contains the NAc, was increased in response to a psychosocial stressor—an effect that could increase drug liking. In another series of studies, researchers examined the relationship among cortisol, dopamine responses to amphetamine, and drug liking in a group of healthy college-age students without a history of alcohol use disorders, drug use, or psychiatric illness. The first of these studies (Oswald et al. 2005) demonstrated that amphetamine-induced dopamine release^5^ correlated with amphetamine-induced cortisol secretion as well as with positive subjective drug responses, such as drug “liking,” “desire,” “good effect,” “high,” and “rush.” The study did not determine, however, whether cortisol responses to a psychological stressor also would be associated with dopamine responses to amphetamine administrated during a separate session. In a second study, the same group of investigators extended these findings by stimulating cortisol secretion through psychological mechanisms (Wand et al. 2007). The researchers employed a test called the Trier Social Stress Test (TSST)— a well-validated procedure that has been widely used to evoke the stress response in human laboratory experiments—to examine whether cortisol responses to psychological stress were associated with enhanced dopamine release and/or subjective responses to amphetamine. The study yielded several important observations:

- Both baseline and stress-induced cortisol levels were positively correlated with dopamine release throughout the ventral striatum, which contains the NAc.
- Cortisol levels were measured for up to 1 month before or after the PET procedures, and the results suggest that the observed relationships between cortisol and dopamine release are not based solely on short-term mechanisms.
- Stress-induced cortisol levels were positively associated with the subjective pleasant effects of amphetamine.

These results suggest that in healthy, young adults without significant prior exposure to AODs, those who secrete high levels of cortisol also release high levels of dopamine and experience greater subjective effects from psychostimulants than those who release only low levels of cortisol and dopamine. The fact that both baseline and stress-induced cortisol levels correlated with dopamine release suggests that ambient cortisol concentrations over time may influence or sensitize mesocorticolimbic dopaminergic signal transmission.

### Altered HPA Axis Dynamics Predates Alcohol Use Disorders

The contention that high and low cortisol states produce clinically meaningful alterations in positive reinforcement of AODs could be supported if altered cortisol dynamics—that is, alterations in the extent and rate at which cortisol levels change after exposure to a stressor—already were found in people who are at increased risk for AOD use disorders (e.g., children of AOD abusers) but have not yet developed these disorders. If aberrant cortisol dynamics predated the development of alcohol dependence and were determined by genetic factors, it should be possible to detect abnormal cortisol responses in the offspring of AOD abusers. Several studies have compared people at increased risk for alcohol use disorders (e.g., children of alcoholics) with people at low risk for these disorders with respect to various characteristics. Using this approach, Schuckit and Smith (1996) found that HPA axis responses study participants exhibited when they were exposed to alcohol predicted future development of alcoholism. Other investigators stimulated the HPA axis using different approaches (King et al. 2002; Oswald and Wand 2004; Waltman et al. 1994) and found a correlation between HPA axis dynamics and risk for alcoholism.^6^ More recently, several studies (Uhart et al. 2006; Zimmermann et al. 2004) demonstrated that when the HPA axis was activated by psychological stressors, people at high risk for alcoholism showed greater cortisol responses than people at low risk.

These findings are not unequivocal, however, because a similar study (Sorocco et al. 2006) of young adult offspring of AOD abusers showed reduced cortisol responses to mental stress. Similarly, an earlier study (Moss et al. 1999) found that cortisol levels measured in the saliva after anticipation stress were lower in high-risk adolescent boys than in control subjects. Indeed, a blunted cortisol response indicates a lack of resilience to stress and may be a form of desensitization resulting from the influence of genes and/or chronic stress on the HPA axis. Moreover, blunted cortisol responses to an acute stressor can signify symptoms of neuroticism and introversion (Oswald et al. 2006)—states in which daily cortisol production can be elevated (Mangold and Wand 2006). Therefore, people with both high and low cortisol responses to acute stress may produce more cortisol per day than people with normal cortisol responses to stress.

## Stage Two: The End of Drug Salience

As described earlier, a reward after drug use that is “greater than predicted” results in increased firing of mesocorticolimbic dopaminergic neurons, whereas a reward that is “less than predicted” results in decreased firing of these neurons (Schultz et al. 1997). During casual use, alcohol and psycho-stimulants stimulate mesocorticolimbic dopamine activity, thereby enhancing drug reward. During the transition from casual use to drug dependence, however, there may be a parallel transition in the reward signal from “better than predicted” to “worse than predicted” (Schultz et al. 1997). As described in this section, glucocorticoids and stress may be involved in this process.

Multiple animal studies have shown that, whereas acute administration of alcohol and psychostimulants increases mesocorticolimbic dopamine (Di Chiara et al. 2004), repeated drug administration followed by abstinence can lower the baseline and stimulated dopamine signal (e.g., Mateo et al. 2005; Zhang et al. 2003).^7^ Additional studies found that the brain reward threshold increases during the transition from the acquisition to the maintenance stage and that this increase correlates with the waning dopamine signal (Koob and Kreek 2007). However, reduced levels of mesocorticolimbic dopamine can be induced not only by repeated drug administration but also by administration of high levels of glucocorticoids (Pacak et al. 2002). This observation suggests a biphasic effect of glucocorticoids on mesocorticolimbic dopamine accumulation, with low levels of glucocorticoids increasing and high levels of glucocorticoids decreasing dopamine accumulation. This conclusion is corroborated by findings that chronic stress can reduce the dopamine signal generated in response to cocaine administration (Gambarana et al. 1999).

In humans, actively drinking, alcohol-dependent people as well as people in acute withdrawal usually generate high levels of cortisol and on occasion actually develop features of hypercortisolism known as the pseudo-Cushing syndrome. Similar activation of the HPA axis also has been observed in psychostimulant users. As in rodents, these elevated cortisol levels may be associated with reduced mesocorticolimbic dopamine activity. This hypothesis is supported by a PET study demonstrating that non–substance abusers who report high levels of life stress have blunted dopamine responses and subjective responses to a single dose of intravenous amphetamine compared with people reporting low levels of life stress (Oswald et al. 2007).

Together, the findings of these animal and human studies show that glucocorticoid levels within a certain range are necessary to generate a normal dopamine signal. It is possible that during the acquisition/preoccupation phase of addiction, glucocorticoids amplify mesocorticolimbic dopamine accumulation in response to AOD administration, thereby enhancing the rewarding experiences associated with drug use. Over time, however, excessive levels of glucocorticoids, along with AOD-induced alterations in glutamate and CRF activity, reduce the dopamine signal, resulting in a decrease in the reward experienced after drug use. This area certainly requires further study.

In summary, sensitization of the reward pathway appears to be fundamental to the transition from AOD use to abuse (Robinson and Berridge 2001). Furthermore, it is plausible that stress and the associated glucocorticoids act as “sensitizers” of the mesocorticolimbic reward pathway during the acquisition/preoccupation stage of AOD use. The amplified dopamine signal, in turn, may boost the reinforcing properties of the drug experience, thereby contributing to vulnerability for transitioning from casual use to abuse. Excessive and/or prolonged stress, however, can produce the opposite result—that is, reward dysfunction. This difference may, in part, be caused by the biphasic properties of glucocorticoids that have a sensitizing effect during early stages of drug exposure but have a lesser effect, or actually impair positive reward, when chronic stress begins to dampen the dopamine signal and induces enhanced expression of CRF. Thus, the animal and human data described here suggest a “Goldilocks” phenomenon, in which cortisol and dopamine levels must not be too high or too low but need to be “just right.”

## Stage Three: Drug Use Based on Negative Affect and Stress

As described above, the positive reinforcing effects of AODs are important in the early stages of drug use but begin to wane as the individual progresses toward addiction. This transition is accompanied by the attenuation or loss of AOD-induced pleasure as well as the development of withdrawal symptoms when AOD use is discontinued (i.e., physical dependence). Both processes lead to a state of negative affect. Negative affect is, in part, a state of internal stress characterized by anxiety, dysphoria, and intense drug craving. Consequently, AOD-dependent people are subjected to both internal stress in the form of negative affect and anxiety and the external types of stress that everybody experiences daily. At this stage, AOD use begins to be motivated primarily by the desire to avoid these negative experiences (i.e., by negative reinforcement).

One brain region implicated in the negative reinforcing effects of both AODs and stress is the extended amygdala, which is located in the medial temporal lobes of the brain and is considered part of the limbic system (see figure 2). The extended amygdala consists of several nuclei with distinct functions. It receives nerve signals from the limbic system and the olfactory system and projects fibers to the hypothalamus and midbrain. The emergence of negative affect during the transition from AOD use to dependence, which is discussed in the following sections, is thought to be driven primarily by neural circuits within the extended amygdala.

### Models of Withdrawal, Negative Affect, and AOD Self-Administration

#### Studies of Rodents

As discussed previously, CRF is a stress-related and anxiety- inducing peptide that is produced in the hypothalamus as well as in other brain regions (e.g., the amygdala). CRF released from the hypothalamus stimulates ACTH secretion and, subsequently, glucocorticoid production. CRF activation in the amygdala, however, stimulates stress and anxiety-like behaviors in rodents and primates, and CRF levels in the amygdala are elevated in these states (Arzt and Holsboer 2006). Consistent with this finding, substances that block the CRF-1 receptor (i.e., CRF-1 receptor antagonists) have been shown to inhibit fear conditioning (Deak et al. 1999) and CRF-induced anxiety-like behaviors (Zorilla et al. 2002).

The contribution of CRF produced outside the hypothalamus to the reinstatement of alcohol drinking after withdrawal (which serves as an indicator of alcohol dependence) has been examined in animal models. Repeated cycles of alcohol exposure and withdrawal can lead to increased alcohol intake at least in certain strains of rodents (e.g., in C57BL/6J mice) (Becker and Lopez 2004; Lopez and Becker 2005). Several studies have implicated the extrahypothalamic CRF system in inducing increased alcohol intake during the withdrawal period in these rodents. For example, Finn and colleagues (2007) found that treatment with a CRF-1 receptor antagonist decreased alcohol drinking in C57BL/6J mice during withdrawal from intermittent alcohol exposure. Other investigators reported that administration of the same CRF-1 receptor antagonist directly into the fluid-filled cavities (i.e., ventricles) of the brain of alcohol-dependent rats reduced the reinstatement of alcohol self-administration following alcohol withdrawal and protracted abstinence (Valdez et al. 2002). This effect was not observed in nondependent rats. Additional studies (Funk et al. 2007; Hansson et al. 2006) using different rodent models and different experimental designs have confirmed these observations. These results suggest that increased CRF activity during alcohol dependence induces motivated alcohol-seeking behaviors that persist into protracted abstinence.

Whether extrahypothalamic CRF affects reinstatement of cocaine self-administration has not been studied as systematically. However, there is substantial evidence that CRF influences psychostimulant as well as opiate self-administration and withdrawal. Several studies (Erb et al. 2005; Maj et al. 2003; Zorrilla et al. 2001) have shown that the levels of CRF mRNA and of some CRF-like protein in the amygdala are altered during self-administration and withdrawal from cocaine and morphine. One study (Richter and Weiss 1999) of cocaine–self-administering rats reported increased CRF in the animals’ amygdala during cocaine withdrawal. Other investigators found that CRF injection into the brains of rats led to reinstatement of cocaine seeking, whereas injection of a CRF-1 receptor antagonist into an area of the amygdala^8^ prevented stress-induced reinstatement of cocaine seeking (Erb et al. 2006). Finally, treatment of rodents with another CRF-1 receptor antagonist significantly attenuated cocaine self-administration, as well as reinstatement of methamphetamine-and morphine-seeking behaviors (Moffett and Goeders 2007; Wang et al. 2006).

#### Human Studies

AOD use disorders have been associated with exposure to both internal and external stressors (Sinha 2007). External stress, such as an unhappy marriage or job dissatisfaction, has been associated with increased alcohol use. Harassment on the job and other work-related stressors also increase the risk of alcohol use disorders. Moreover, in alcohol-dependent people, external stress is an important precipitant of relapse (Sinha et al. 2006). Thus, alcoholics experiencing highly threatening or chronic psychosocial stress following treatment are more likely to relapse than alcoholics not experiencing such stress (Noone et al. 1999).

Other studies have shown that internal stress states (e.g., anxiety) also can be associated with AOD use. A recent study (Marquenie et al. 2007) of more than 7,000 Europeans retrospectively and prospectively analyzed the nature of the relationship between co-morbid alcohol dependence and anxiety disorders, which can be considered a form of chronic stress. Alcohol dependence was associated with high rates of lifetime anxiety; moreover, anxiety generally preceded the development of alcohol use disorders. Another large-scale, international study investigating patterns of comorbidity between substance use and psychiatric disorders in six nations in the Americas, Europe, and Japan also suggested that the presence of an anxiety disorder may predispose to the development of AOD use disorders (Merikangas et al. 1998). In that study, across all sites, 45 percent of participants with drug dependence also met the criteria for an anxiety disorder. Importantly, in contrast to the affective disorders (e.g., depression), which typically developed following the onset of AOD problems, the onset of anxiety disorders preceded AOD use disorders at nearly all levels of severity of substance use disorders. This finding raises the possibility of a causal link between anxiety and vulnerability to the development of AOD use disorders. Finally, Terra and colleagues (2006) examined the relationship between social phobia and alcohol use in 300 hospitalized alcoholic patients. The study confirmed the high prevalence of anxiety disorders, particularly social phobia, among alcoholics; moreover, the results demonstrated that in most cases social phobia preceded alcohol dependence.

Several studies have focused on people with a dual diagnosis of combat-related posttraumatic stress disorder (PTSD) and AOD abuse (Donovan et al. 2001). Although a causal relationship between exposure to combat-related stress and AOD abuse has not been clearly established, veterans with PTSD typically report a higher lifetime use of alcohol, cocaine, and heroin than veterans screening negative for PTSD (Saxon et al. 2001).

Human laboratory studies have corroborated these findings. For example, Fox and colleagues (2007) demonstrated that in treatment-seeking alcohol-dependent people, exposure to stress imagery significantly increased alcohol craving, anxiety, and negative emotions. Similar observations were made in cocaine-dependent patients undergoing treatment (Fox et al. 2007). The same group of investigators also reported that higher levels of stress-induced cocaine craving were associated with a shorter time to relapse and that stress-induced levels of ACTH and cortisol predicted higher amounts of cocaine used per occasion over a 90-day followup (Sinha et al. 2006). Brain imaging studies have begun to identify the brain structures involved in stress-and drug cue–induced craving states. Findings indicate considerable overlap between the neural circuits that process cues signaling stress and drug use, with activity in the corticostriatal limbic circuitry underlying both affective and reward processing (Sinha and Li 2007).

Researchers have long sought to explain why various forms of external stress may precede the development of AOD use disorders and precipitate relapse. Even before it was studied systematically, this association was first explained by the tension reduction hypothesis proposed by Conger and colleagues (1956). According to this hypothesis, the reduction of negative feelings by drinking alcohol may represent an important reinforcing effect. In fact, many studies using a variety of experimental strategies have demonstrated that alcohol can dampen the stress response (Sayette 1999). In general, these studies have demonstrated that people considered at risk for developing alcohol-related problems exhibit attenuation of stress reactions in psychologically challenging experimental sessions after receiving alcohol (Sinha et al. 1998). Indeed, this may be one possible mechanism by which a positive family history increases the risk for alcoholism. However, numerous other individual differences and situational factors affect the extent to which a person experiences stress-response dampening after consuming alcohol. These factors include, for example, personality traits, extent of self-consciousness, cognitive functioning, and gender (Sher et al. 2007).

### Mechanism Underlying the Development of Negative Affect

#### HPA Axis

Drug-induced activation of the HPA axis may sensitize reward pathways early on in the addiction process. Chronic drug use, however, results in gross impairments of the HPA axis that may injure reward pathways and activate CRF and other stress factors during heavy AOD use (i.e., bingeing) and withdrawal.

For more than two decades, numerous studies have shown that cortisol dynamics are impaired in alcohol-dependent people compared with nonalcoholics (Wand 1993). In rodents, long-term daily administration of alcohol in a liquid diet decreased HPA axis activity, and this reduction persisted up to 3 weeks postabstinence (Rasmussen et al. 2000). In humans, the characteristics of the abnormalities in the HPA axis depend on whether the alcohol-dependent person is studied during intoxication, when experiencing various stages of withdrawal, or after being abstinent for several days or months. As mentioned earlier, actively drinking, alcohol-dependent people as well as people in acute withdrawal usually generate high levels of cortisol or even exhibit clinically relevant hypercortisolism. Once withdrawal symptoms have resolved, however, most alcoholics enter a phase characterized by an impaired ability to generate normal amounts of cortisol in response to pharmacological and psychological challenges (Adinoff et al. 2005; Wand 1993). Thus, various stages of AOD use are associated with both high and low cortisol states. Moreover, high cortisol levels during early drug exposure may increase drug taking, whereas low cortisol levels during abstinence may help precipitate relapse.

A similar pattern of HPA axis activation and then injury has been observed in both rodents and humans exposed to cocaine. In rodents, acute and repeated cocaine administration initially activates the HPA axis, resulting in elevated corticosterone release. With repeated high-dose cocaine administration over time, however, corticosterone levels progressively decline (Koob and Kreek 2007). Similar findings have been obtained in human long-term cocaine addicts in a clinical laboratory setting (Aouizerate et al. 2006).

### Extended Amygdala

As mentioned earlier, the extended amygdala consists of several nuclei with distinct functions. These include the basolateral amygdala, the central and medial amygdala, and the cortical nucleus. Several stress peptides and neurotransmitters associated with the extended amygdala appear to influence the development of negative affect and anxiety. As described above, CRF is an anxiety-inducing neuropeptide. Studies in rats identified increased extracellular levels of CRF in the central amygdala during acute alcohol withdrawal and during exposure to various other forms of stress (Merlo-Pich et al. 1995). Administration of CRF antagonists reversed anxiety-like behaviors as well as excessive alcohol drinking associated with alcohol withdrawal (Valdez et al. 2003). Other studies have implicated nerve signal transmission mediated by the neurotransmitter γ-aminobutyric acid (GABA) in the central amygdala in regulating alcohol intake (Hyytia and Koob 1995). Chronic alcohol administration is associated with increased GABA release in the central amygdala, in part in response to CRF.

Another signaling molecule that can modulate anxiety and alcohol abuse through its actions on the amygdala is called neuropeptide Y (NPY) (Valdez and Koob 2004). Various measures to disrupt normal NPY function all result in anxiety-like behaviors and increased alcohol consumption (Thiele et al. 1998; Thiele and Badia-Elder 2003). NPY production is in part controlled by a regulatory factor known as cAMP-responsive element–binding protein (CREB). Mice in which the CREB protein has been inactivated have a higher preference for alcohol than do normal mice (Pandey et al. 2004). Similarly, the levels of both NPY and the activated form of CREB are reduced in the amygdala of alcohol-preferring (P) rats compared with nonpreferring (NP) rats (Suzuki et al. 2004). Interestingly, the selectively bred P rats not only consume greater amounts of alcohol but also have higher baseline levels of anxiety-like behaviors compared with NP rats (Kampov-Polevoy et al. 2000). One hypothesis is that the P rats drink excessive amounts of alcohol in order to reduce anxiety levels. This hypothesis is supported by findings that alcohol stimulated a signaling mechanism called the cAMP/protein kinase A (PKA) pathway in the central and medial amygdala (but not the basolateral amygdala) that led to increased levels of several regulatory molecules, including NPY and CREB function (Pandey et al. 2005). These changes were accompanied by a reduction in anxiety-like behavior in the P rats. Moreover, infusion of a molecule that can activate these signaling mechanisms or of NPY directly into the central amygdala reproduced the neurochemical and anxiety-reducing effects of alcohol. These effects were observed only in P rats, not in NP rats. These findings show that decreased CREB function in the central amygdala is associated with maintaining high anxiety levels and alcohol-drinking behaviors of P rats.

Another group of potential amygdala modulators are small molecules called cocaine- and amphetamine-regulated transcript (CART) peptides, which attenuate the behavioral effects of cocaine (Jaworski and Jones 2006). These peptides are thought to modulate neural activity in the amygdala and other CRF-rich brain regions. CART peptides stimulate CRF and glucocorticoid release, while at the same time CRF and glucocorticoids increase the production of the CART peptides. Stress and psychostimulant exposure modulate CART expression in the hypothalamus and amygdala through cAMP/PKA and CREB signaling. However, the role of CART in drug-induced negative affect states needs further investigation.

Thus, an extensive regulatory network in the amygdala, comprising NPY, CRF, GABA, CREB, the cAMP/PKA signaling pathway, and possibly CART peptides, may influence stress-induced anxiety and drug intake. To date, it is unclear, however, if and how glucocorticoids alter these systems. Additionally, this currently known network may just be the “tip of the iceberg,” and other regulatory mechanisms may have an impact as well.

## Conclusion

The relationships between stress and AOD use disorders are complex. Prevailing evidence suggests that causality exists in both directions: Internal and external stress can enhance AOD use and AOD addiction creates an internal state of stress as part of the negative-affect state. The generalizations described in this review oversimplify a field that requires further intensive investigation. However, several salient points can be made.

**Figure 4:**
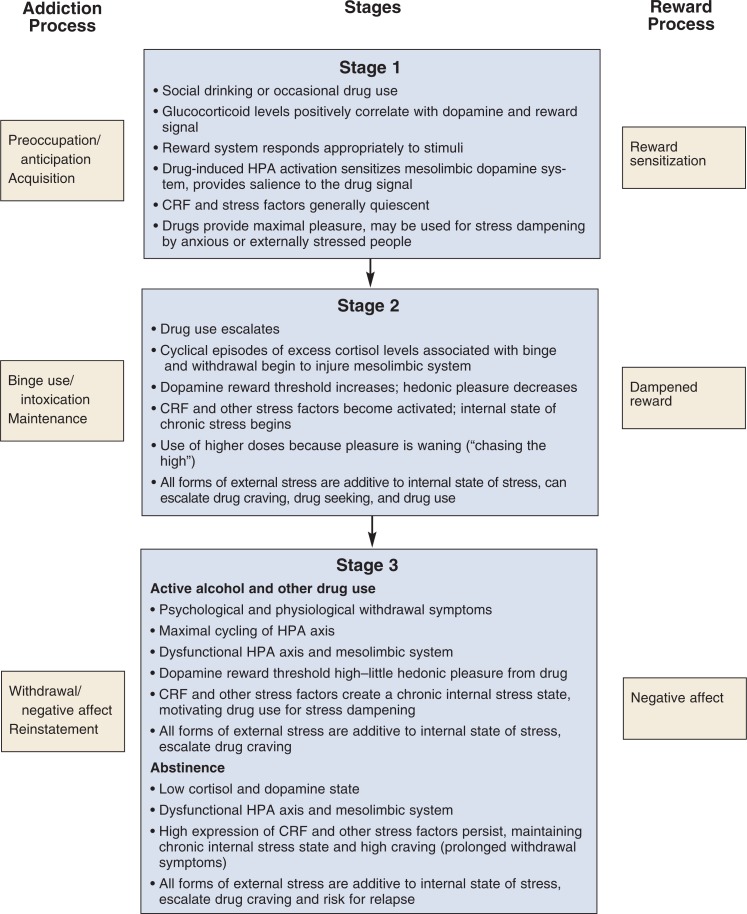
Changes in the body’s response to stress and the reward system over the three stages of the drug addiction process. The stages of the addiction process are shown on the left, the state of the body’s reward system is indicated on the right. NOTES: CRF = corticotropin-releasing factor; HPA = hypothalamic–pituitary–adrenal axis.

Both stress and AODs activate the HPA axis and extended amygdala in a time- and dose-dependent manner. Animal and human studies have generally shown that specific forms of stress are associated with increased AOD use and can precipitate relapse. The effects of stress on AOD use depend in part on the type, context, and severity of the stressor; the genetic background of the AOD user; and the interplay between the positive and negative reinforcing influences of the drug and the stressor. Early in the development of AOD use, both stress- and drug-induced activation of the HPA axis allow glucocorticoids to sensitize the reward pathways, although the exact effects on the mesocorticolimbic dopamine system still are unknown. As a person transitions from casual AOD use (e.g., social drinking) to AOD dependence, the balance between the positive and negative reinforcing effects of the drug shifts (see figure 4). Although multiple brain regions are involved in this transition, the mesocorticolimbic dopamine system (which is modulated by various forms of stress, glucocorticoids, and neuroactive steroids) and the extended amygdala (through alterations in CRF, NPY, CREB, and GABA) likely alter the reinforcing properties of both AODs and stress. With the appearance of drug dependence and episodes of withdrawal, the neurochemical environment in the amygdala shifts to produce anxiety-inducing states by increasing expression of CRF and further activating the HPA axis. This neurochemical milieu creates craving and drug-seeking behaviors.

The hypothesis that the stress response and the risk of AOD abuse and dependence are interrelated is supported by the observation that the offspring of alcohol-dependent people, who are at increased risk for alcohol use disorders, have an altered cortisol response to various stressors that precedes the onset of heavy drinking. Thus, a dangerous interaction between stress and drug-seeking behaviors likely exists throughout the different stages of addiction, aided by the allostatic adaptive mechanisms described in this review.

Understanding the allostatic adaptive process and its interaction with stress and the risk of AOD abuse and dependence will someday enable researchers and clinicians to target specific treatment interventions to patients at a specific stage of addiction. For example, ongoing pharmacological research is aimed at developing medications that modulate the activity of glucocortioids, CRF, NPY, and the other stress-regulated factors that are involved in the transition from AOD use to AOD dependence. Most of these studies still are in a preclinical stage but will soon advance to human trials. But although these exciting interventions still are many years away, important information already can be shared with high-risk populations. For example, children of alcoholics, with their high cortisol responses to stress, are at increased risk for alcohol abuse and dependence. These people should be made aware of the biological reasons that place them at higher risk for addiction and should be counseled to limit or avoid alcohol. Patients with PTSD and other anxiety disorders constitute another high-risk population and should be educated accordingly about the dangers of using AODs to self-medicate prior to social interactions. Finally, any treatment plan for addicted patients must be fluid and periodically reevaluated to take into account the emergence of internal and external stressors that could precipitate relapse.

## Footnotes

1 For a definition of this and other technical terms, see the glossary on pp. 177–179

2 CRF sometimes also is called corticotrophin-releasing hormone (CRH).

3 This is called a biphasic effect.

4 These differences are not related to the duration of the stressful situations (i.e., whether acute, repeated, or chronic stress was studied).

5 Dopamine release was defined as amphetamine-induced changes in the ability of the dopamine antagonist raclopride to bind to dopamine receptors. If high levels of dopamine are released, all receptors are occupied by the dopamine and raclopride cannot bind. If dopamine release declines, however, more receptors become unoccupied and the ability of raclopride to bind to these receptors increases.

6 Waltman and colleagues (1994) used CRF administration to stimulate the HPA axis. Oswald and Wand (2004) and King and colleagues (2002) used opioid receptor antagonists to stimulate the HPA axis. Opioids are brain-signaling molecules that modulate nerve signal transmission, thereby affecting numerous processes in the brain. Among others, opioid-mediated nerve signals inhibit CRF-releasing neurons, thereby preventing CRF from acting on the HPA axis. Opioid receptor antagonists will block this inhibition and thus activate the HPA axis.

7 Several PET analyses identified an analogous situation in humans, because the mesocorticolimbic dopamine system was downregulated in alcohol-dependent people as well as in chronic users of cocaine and methamphetamine (Volkow et al. 2003).

8 The CRF-1 receptor antagonist was injected into the bed nucleus of the stria terminalis.
